# The prevention of musculoskeletal complaints: long-term effect of a work-related psychosocial coaching intervention compared to physiotherapy alone—a randomized controlled trial

**DOI:** 10.1007/s00420-020-01538-1

**Published:** 2020-04-09

**Authors:** Annette Becker, Peter Angerer, Jeannette Weber, Andreas Müller

**Affiliations:** 1 Heinrich-Heine-University Düsseldorf, Düsseldorf, Germany; 2 Institute of Psychology, Work and Organizational Psychology, University of Duisburg-Essen, Essen, Germany

**Keywords:** Long-term effect, Stress prevention, Musculoskeletal complaints, Nurses, Coaching, Selection, optimization, and compensation

## Abstract

**Purpose:**

Research shows that psychosocial factors play a significant role in the emergence of musculoskeletal complaints (MSC). The aim of this study was to determine the long-term effects on unspecific MSC by a combined physiotherapy and coaching intervention compared to physiotherapy alone. The coaching intervention focussed on enabling better strategies for coping with work stressors.

**Methods:**

The participants of a previous randomized controlled intervention were invited to participate again in a third follow-up survey 22 months after the end of the intervention. In 2014, 65 nurses completed a 10-week personalised physiotherapy. Additionally, the intervention group (*n* = 33) passed five individual coaching sessions, plus an opening and closing session. 44 nurses (IG: *n* = 24; CG: *n* = 20) passed again a physical examination as well as another questionnaire assessment in 2016. The primary outcome was MSC, secondary outcomes were work ability and work-related well-being. Due to missing data, multiple imputations were conducted using the mice package in R. Data were analysed by ANOVA with two-way repeated measures, *t* tests for independent samples and Chi-squared tests.

**Results:**

In respect of MSC, stronger improvement of movement in the vertebral column was observed in the IG compared to the CG. No differences between the IG and CG regarding other long-term effects were observed.

**Conclusions:**

The results suggest that the combined intervention of work-related coaching and physiotherapy had only a marginally stronger long-term effect with respect to MSC than physiotherapy alone.

## Introduction

One of the main causes of sickness absence worldwide is musculoskeletal complaints (MSC), such as head, shoulder, neck or back pain (Edwards and Greasley 2010; Luttmann et al. 2003; WHO 2017). Population studies have shown a lifetime prevalence of 60–80% for back pain (e.g. Campbell et al. 2013; Cote et al. 1998; Papageorgiou et al. 1995). It is expected that in 80–85% of cases of MSC, the aetiology is unspecific. Hence, only a small proportion of MSC is related to a defined pathology, such as tumors, infections, or osteoporosis (e.g. Abedini et al. 2014; Kool et al. 2004).

Besides physical stress factors (e.g. Burdorf and Sorock 1997; Engels et al. 1996; Lorusso et al. 2007; WHO 2018), psychosocial factors play a significant role in emergence and persistence of MSC (e.g. Auvinen et al. 2017; da Costa and Vieira 2010; Harcombe et al. 2010; Linton 2000; Sadeghian et al. 2014). It is assumed that persistent psychosocial stress can contribute to biomechanical strain on the locomotor system, to inadequate recovery of stress-activated muscles, and to degenerative processes (e.g. Geurts and Sonnentag 2006; Griffiths et al. 2007; Larsman et al. 2013; Lundberg 2002). Studies in the work context indicate that psychosocial work stressors (e.g. high job demands, insufficient job control, low social support) are related to the occurrence and persistence of MSC (e.g. Bernal et al. 2015; Campbell et al. 2013; da Costa and Vieira 2010; Hoogendoorn et al. 2000; Lang et al. 2012; Melloh et al. 2013; Sterud et al. 2014). Those findings suggest that the prevention of unspecific MSC should include work-related aspects such as psychosocial work stressors (e.g. Airaksinen et al. 2006; Sembajwe et al. 2013).

However, only few intervention studies to prevent MSC have been conducted so far, which also took psychosocial work stressors into account and compared the effects of these approaches with standard physiotherapy programs alone (e.g. Ewert et al. 2009; Horneij et al. 2001). In one study, a multimodal prevention program (containing cognitive behavioural therapy aspects and stress-reducing techniques in addition to physiotherapy) showed no superior effects regarding back, shoulder and neck pain intensity and functioning compared to a physiotherapy program alone (Ewert et al. 2009). In another study, Horneij et al. (2001) compared three groups of which one passed an individually designed physical training programme (group 1), the second one a work-place stress management programme (group 2) and the third one acted as a control group receiving no intervention (group 3). The results revealed no significant differences between the three groups regarding MSC, perceived physical exertion and work-related psychosocial factors.

Moreover, research concerning long-term effects of interventions to reduce MSC is generally still pending (e.g. van Poppel et al. 2004, Tveito et al. 2004). Follow-up examinations of intervention effects on MSC at work are mostly performed just after 6–12 months after the intervention and very rarely after eighteen months or more (e.g. van Poppel et al. 2004).

According to our knowledge, there is a lack of studies, which determined the long-term effects of interventions to reduce MSC in the work context. and which took both psychosocial aspects and physiotherapy into account. Only the study of Horneij et al. (2001) observed a tendency of improvement in low back pain up to 18 months after an individually designed physical training program as well as after a work-place stress management intervention among home-care personnel.

Further findings, with regard to long-term intervention effects on MSC, are inconsistent. In one study, the prevalence of low back pain episodes increased from baseline to follow-up (after 24 months) among hospital staff receiving no intervention, but did not increase among hospital staff receiving an educational program (safe postures and patient handling, advice by educators). Furthermore, the number of sick leaves longer than 30 days decreased significantly in this intervention group (Fanello et al. 1999).

A long-term study focussing on preserving work ability has shown that group-based, vocationally oriented multidisciplinary early rehabilitation (VOMR) was not associated with a lower risk of long-term work disability after 2.8 years when compared to a control group (no rehabilitation). The VOMR included three or four periods (in total 15–21 days) of in-patient extensive, multi-professional rehabilitation for almost healthy persons combining stress-management physical training with ergonomic education (Saltychev et al. 2012). Other studies investigating long-term changes in work ability after passing interdisciplinary rehabilitation of chronic musculoskeletal disorders (comparing two multidisciplinary inpatient rehabilitation programmes for patients with fibromyalgia) did not find effects on perceived work ability (Saltychev et al. 2014; Suoyrjo et al. 2009).

Against this background, the aim of the present randomised controlled study was to determine the long-term effects of a psychosocial coaching intervention for nurses, which focussed on coping with psychosocial work stressors. More specifically, we aimed to investigate whether the psychosocial coaching intervention combined with physiotherapy (physical exercise) is superior to physiotherapy alone as a standard intervention to reduce unspecific MSC. Results on the short-term effects of this randomised controlled study have been reported elsewhere (Becker et al. 2017). To summarise, the results on short-term effects suggest that this psychosocial coaching intervention can contribute to the reduction of MSC, improvement of work ability, and work-related well-being up to 3 months after the end of the intervention. In this report, we present the results of a follow-up survey that was realized 22 months after the end of the intervention (24 months after the start of the intervention) to determine the long-term effect of psychosocial coaching on the functional status of the locomotor system, pain, work ability and work-related, and psychological well-being.

## Methods

### Study design

In a previous randomised controlled study in 2014 (Becker et al. 2017), 68 registered nurses were randomised to an Intervention Group (IG) or Control Group (CG). The primary aim was to reach a total sample size of 80 participants (40 per study group). With a power of 0.80, an alpha level of 0.05, and a mean correlation of *r* = 0.60 between the repeated measurements of the target variables, the proposed sample size should enable to test weak effects (*f* = 0.28) of the intervention. This targeted sample size of 80 participants was not met mainly because high workload and high coordination efforts in the cooperating hospitals hampered the participation of the nurses. Moreover, 27 nurses were excluded at baseline as they either did not meet the inclusion criteria or had lost their initial interest in the study.

All 68 participants worked in a hospital, were not off work due to illness at the time of the study and had experienced non-specific MSC of the locomotor system at the time of the examination. Exclusion criteria included specific physical symptoms or no MSC at the time of the examination, serious other illnesses and current participation in other medical or therapeutic treatment. An initial physical examination especially of the motor system was performed to identify those exclusion criteria. After this initial examination and admission to the study, the participants drew a lot (simple randomization) out of a not-observable vessel and were thus assigned to the IG and CG.

Both groups received physiotherapy exercises during a period of ten weeks (focussing on individual functional status and specific physical job demands; 10 × 45 min). Additionally, the IG joined a work-related psychosocial coaching intervention during this period. This psychosocial coaching intervention consisted out of 1 × 120-min introduction to the theoretical model of selection, optimization and compensation (SOC, Baltes and Baltes 1990), and 5 × 90-min stress intervention coaching based on the SOC model, i.e. participants developed an individual goal to reduce work stress or to improve resources at work by means of optimized and compensatory problem-solving strategies and self-reflection. The coaching was carried out by the first author (A.B.) as a certified supervisor and coach (German association for supervision—DGSv), management consultant (M.A.), physiotherapist and teacher. Experienced licensed physiotherapists accompanied the physiotherapy and conducted the examination.

The outcome parameters were assessed by physical examinations and by validated questionnaire tools. Physical examinations were performed at the beginning of the study (baseline) and immediately after the end of the intervention (1st follow-up, 11 weeks after the start of the intervention). The questionnaires were used at the beginning, on five occasions during, at the end and twelve weeks after the end (2nd follow-up, 22 weeks after the start of the intervention) of the intervention phase.

In the present study, we conducted a 3rd follow-up survey (physical examination as well as a final questionnaire assessment) in the IG and the CG, scheduled 24 months after the start of the intervention. We chose this time interval for the following reasons: First, there is a general lack of studies investigating long-term effects of MSC interventions at work (e. g. van Poppel et al. 2004). However, for therapeutic and health economic reasons, such studies are necessary to assess the sustainability of the effectiveness of these interventions. Second, the few studies on long-term effects known to us covered time intervals of 18 to about 34 months (Fanello et al. 1999; Horneij et al. 2001; Saltychev et al. 2012). We have decided to carry out our follow-up study within this time interval, to enable comparability of our findings with those previous studies. All data were collected between March 2016 and October 2016.

### Participants

Acquisition of participants took place from March 2016 to September 2016. All 65 nurses, who completed the previous study in 2014, were invited by letter to participate in a follow-up study. The nurses confirmed their participation or non-participation again by letter. In some cases, phone calls were desired to resolve personal questions (e.g. illness). Criteria which led to exclusion from the follow-up study were serious other illnesses. Forty-four nurses participated in the follow-up examination. Two persons were excluded and 19 participants had lost interest to participate in the study or were lost to follow-up due to personal reasons like relocation, change of employer or family affairs.

### Measures

#### MSC

*Functional status of the locomotor system *Mobility restrictions and restrictions of muscle strength were assessed by a screening examination according to Spallek et al. (2007). The examination according to Spallek is recommended by the German Federal Institute for Occupational Safety and Health (BAuA) in the context of the diagnostics of MSC in occupational health practice (Grifka et al. 2005) and has already been implemented several times in research (e.g. Heiden et al. 2013). Elements of the systematic orthopaedic medical examinations according to Cyriax (e.g. Atkins et al. 2010; Cyriax 1982; Cyriax and Coldham 1984; Cyriax and Cyriax 1996; Ombregt 2013) supplemented Spallek’s assessments with reference to the status of the joints, muscles and peripheral nerves. The standardised assessment was aligned with the norms set down by Spallek (extent of movement: neutral-zero-measurement, distance details: cm) and on structured procedures by Cyriax (active, passive, isometric tests). The physiotherapists who carried out the examination were blinded against the allocation of participants to the study group. Each participant completed the baseline and 1st follow-up examination in 2014 as well as the 3rd follow-up examination in 2016 with the same physiotherapist.

Functional status of the locomotor system in everyday life was assessed by one scale of the Nordic Musculoskeletal Questionnaire (NMQ) (Kuorinka et al. 1987), assessing restrictions in everyday activities due to MSC in nine regions of the body. The NMQ has already been used for the capture of musculoskeletal complaints and symptom-related restrictions in the ability to work in the field of nursing (Trinkoff et al. 2002).

*Pain severity and impairment by pain *Pain at the end-of-range movements (maximum degree movements) was assessed during the physical examinations. Pain severity during everyday movements and impairments due to pain in everyday life were measured by the relevant two subscales (pain severity, impairment due to pain) of the West Haven–Yale Multidimensional Pain Inventory (WHYMPI) (Kerns et al. 1985).

#### Work ability

Work ability was assessed using three items of the German Version of the Work Ability Index [WAI; Hasselhorn and Freude (2007)]. The WAI is a well-established tool to measure work ability (Tuomi et al. 1998, 2001). Studies have shown that the WAI is predictive for sickness absence and health-related quality of life (e.g. Ahlstrom et al. 2010) as well as ambitions for early retirement (von Bonsdorff et al. 2010). We used the first and second dimensions of the WAI. The first dimension queries current work ability compared with lifetime best and the second dimension queries current work ability with reference to physical and psychological job demands.

#### Work-related psychological well-being

Work-related psychological strain was measured by the German version of the Irritation Scale (Mohr et al. 2006). It consists of eight items, addressing perceived emotional and cognitive strains in occupational contexts with reference to ruminative thoughts (cognitive irritation) on work-related themes and irritated reactions (emotional irritation). The scale has been proven to be reliable and valid in various studies (Mohr et al. 2005).

As an additional indicator for the impairment of mental well-being, burnout was captured by the Maslach Burnout Inventory [MBI; Maslach and Jackson (1981)]. MBI is the most frequently applied scale in burnout-research (Hedderich 2009). In this study, a short version of the MBI was used with three items each for emotional exhaustion and depersonalisation.

### Analyses

Due to missing data, multiple imputation by chained equations was conducted using the *mice* package in R (Multivariate Imputation by Chained Equations in R; van Buuren and Groothuis-Oudshoorn 2011). The dataset with all variables including baseline and each follow-up was used assuming missing at random (MAR). For imputation, predictive mean matching was chosen with automatic generation of a predictor matrix. IG was always included as a predictor when missing values were calculated. Furthermore, the variables were imputed in ascending order of the number of missing values. Imputation of missing data was conducted generating 20 complete data sets.

Further analysis was carried out using IBM SPSS Statistics 24.

To analyse intervention effects on the outcomes, two-way repeated measures ANOVA was conducted with time as the within-subject variable and IG as the between-subject variable. Time × group interaction effects indicate intervention effects, where differences in improvement of the study variables between the intervention and control group occurred. For imputed datasets, the procedure as described by van Ginkel was used (van Ginkel 2016a). In more detail, two-way repeated measures ANOVA was carried out for each imputed dataset separately. Within those analyses, time and intervention groups were effect coded and handled as fixed effects. Parameter estimates as well as between-imputation and within-imputation covariance matrices of those analyses were then used to obtain pooled *F* tests. Pooled *F* tests were performed using the SPSS syntax by van Ginkel (2016b). The number of levels of the independent variables was specified as 1 for time, intervention group and their interaction. To specify the number of levels of the dependent variable and intercept, the default options of 1 for continuous variables were used. Furthermore, the standard approximation of degrees of freedom was used.

To analyse differences between participants and drop-outs, *t* tests for independent samples or Chi-squared tests were conducted. Statistical significance was assumed at *p* < 0.05.

## Results

### Sample description and drop-out analyses

**Fig. 1:**
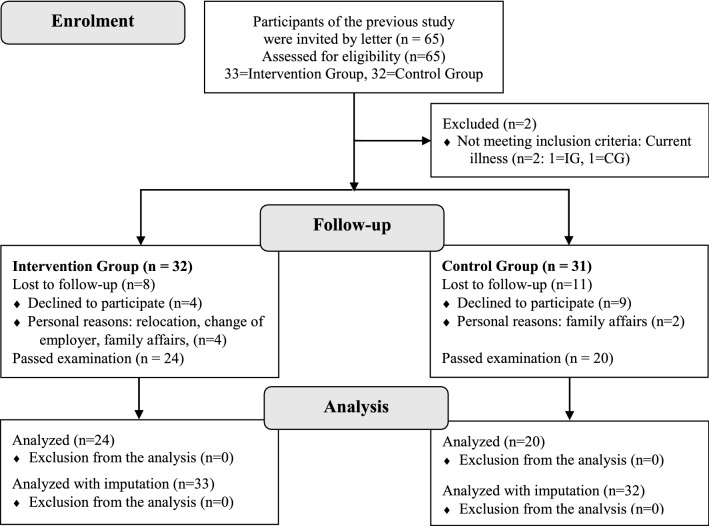
Recruitment of the participants

Forty-four nurses registered to participate in the 3rd follow-up survey (see Fig. 1).

Two of the 65 initially invited nurses were excluded before the beginning of the survey because of current illness. Nineteen persons lost their interest in the study or withdrew due to personal reasons (e.g. relocation, change of employer, family affairs). The remaining 44 nurses passed the examination (IG:* n* = 24; CG: *n* = 20) and fully completed the survey. However, due to multiple imputation of missing data, all 65 nurses were included in the analyses. For the 3rd follow-up, values of restriction of muscle strength, restriction of maximum degree movement and pain due to maximum degree movement were missing in 21 cases, for pain severity, impairment due to pain and burnout in 22 cases and for work ability and irritation in 23 cases. No values were missing for baseline variables. In 20 cases, all outcome variables were missing and in one case only the work ability or irritation variables were missing, respectively. In one further case, all outcome variables were missing except for restriction of muscle strength, restriction of maximum degree movement and pain due to maximum degree movement.

At baseline, no significant differences were detected between the participants of the 3rd follow-up survey and the drop-out group in respect to important demographic characteristics (see Table 1).

**Table 1:** Demographic variables—sample description and drop-out analyses

	Participants at 2nd follow-up (*N* = 65)	Participants at 3rd follow-up (*N* = 44)	Drop-outs (*N* = 21)	*T *(*df* = 63)
	*M* (SD)	*M* (SD)	*M* (SD)	
Age (years)	43.66 (10.34)	43.98 (9.59)	43.00 (11.99)	0.354 (n.s.)
Employment in nursing (years)	23.05 (10.23)	23.64 (9.74)	21.81 (11.34)	0.670 (n.s.)
	%	%	%	*χ*^2^ (*df* = 1)
Gender (female)	86.15	86.36	85.71	0.005 (n.s.)
Employed (full-time)	47.69	40.91	61.90	2.512 (n.s.)
Employed (indefinite contract)	98.46	100.00	95.24	2.128 (n.s.)
Nurses in a senior position	20.00	20.45	19.05	0.018 (n.s.)

*M* arithmetic mean, *SD* standard deviation

86% of nurses who took part in this follow-up study were women. Nurses were on average 44 years old and had worked in the nursing profession for about 24 years. The proportion of nurses in leadership positions was about 20%. Less than half of the participants (41%) worked full-time. The number of full-time working nurses in the drop-out group was 62%.

Participants of the 3rd follow-up and the drop-out group did not differ regarding MSC, work ability, and work-related well-being at baseline (see Table 2).

**Table 2:** Outcomes—sample description and drop-out analyses

		Total (*N* = 65)	Participants at 3rd follow-up (*N* = 44)	Drop-out (*N* = 21)	*t* (*df* = 63)
		*M* (SD)	*M* (SD)	*M* (SD)	
*Primary outcome*					
MSC					
Functional status of the locomotor system					
FE	Restriction of muscle strength (E) (range 0–12)	4.97 (2.96)	4.98 (2.94)	4.95 (3.09)	− 0.031 (n.s.)
	Restriction of maximum degree movement (E) (vertebral column) (range 0–24)	6.00 (4.66)	6.30 (4.55)	5.38 (4.93)	− 0.737 (n.s.)
NMQ	Restriction of everyday activities (last 3 months) (range 0–9)	1.15 (1.77)	1.16 (1.64)	1.14 (2.06)	− 0.034 (n.s.)
Pain severity/impairment due to pain					
FE	Pain due to maximum degree movement (range 0–210)	14.95 (17.76)	14.68 (16.21)	15.52 (21.08)	0.177 (n.s.)
WHYMPI	Pain severity on everyday movements (range 0–6)	2.40 (1.13)	2.30 (1.12)	2.60 (1.13)	1.004 (n.s.)
	Impairment due to pain (range 0–6)	2.01 (1.22)	1.96 (1.33)	2.12 (0.95)	0.515 (n.s.)
*Secondary outcome*					
Work ability					
WAI	Current work ability compared with lifetime best (range 0–10)	7.08 (1.86)	7.05 (1.95)	7.14 (1.68)	0.196 (n.s.)
	Work ability with respect to physical job demands (range 1–5)	3.62 (0.68)	3.59 (0.69)	3.67 (0.66)	0.419 (n.s.)
	Work ability with respect to mental job demands (range 1–5)	3.65 (0.86)	3.55 (0.90)	3.86 (0.73)	1.492 (n.s.)
	Work ability with respect to physical and mental job demands (range 2–10)	7.26 (1.28)	7.14 (1.30)	7.52 (1.21)	1.146 (n.s.)
Work-related, psychological well-being					
Irritation	Cognitive irritation (range 1–7)	3.85 (1.63)	3.93 (1.63)	3.67 (1.65)	− 0.612 (n.s.)
	Emotional irritation (range 1–7)	3.07 (1.44)	3.16 (1.50)	2.89 (1.31)	− 0.714 (n.s.)
	Overall irritation (range 1–7)	3.36 (1.38)	3.45 (1.39)	3.18 (1.37)	− 0.737 (n.s.)
MBI	Emotional exhaustion (range 1–6)	3.28 (1.14)	3.29 (1.18)	3.27 (1.07)	− 0.059 (n.s.)
	Depersonalisation (range 1–6)	1.67 (0.77)	1.64 (0.80)	1.71 (0.72)	0.342 (n.s.)

*E* external assessment, *FE* functional examination, *M* arithmetic mean, *MBI* Maslach Burnout Inventory, *MSC* musculoskeletal complaints, *NMQ* Nordic Musculoskeletal Questionnaire, *SD* standard deviation, *WAI* Work Ability Index, *WHYMPI* West Haven–Yale Multidimensional Pain Inventory

Further analysis in respect of intervention effects at the 2nd follow-up on MSC, work ability, and work-related well-being showed almost no differences between participants and drop-outs. A significant difference between the two groups was only observed for emotional exhaustion. Within the drop-out group, greater improvement in emotional exhaustion was found at the 2nd follow-up (see Table 3).

**Table 3:** Changes in outcomes from baseline to 2nd follow-up—sample description and drop-out analyses

		Total (*N* = 65)	Participants at 3rd follow-up (*N* = 44)	Drop-out (*N* = 21)	*t* (*df* = 62)
		*M* (SD)	*M* (SD)	*M* (SD)	
*Primary outcome*					
MSC					
Functional status of the locomotor system					
FE	Restriction of muscle strength (*E*) (range 0–12)	− 1.00 (2.57)	− 1.05 (2.12)	− 0.90 (3.42)	0.208 (n.s.)
	Restriction of maximum degree movement (*E*) (vertebral column) (range 0–24)	− 2.42 (3.95)	− 2.25 (4.15)	− 2.80 (3.52)	− 0.514 (n.s.)
NMQ	Restriction of everyday activities (last 3 months) (range 0–9)	− 0.64 (1.35)	− 0.75 (1.40)	− 0.40 (1.23)	0.961 (n.s.)
Pain severity/impairment due to pain					
FE	Pain due to maximum degree movement (range 0–210)	− 8.48 (14.38)	− 9.16 (13.64)	− 7.00 (16.16)	0.554 (n.s.)
WHYMPI	Pain severity on everyday movements (range 0–6)	− 0.59 (1.14)	− 0.64 (1.18)	− 0.50 (1.06)	0.442 (n.s.)
	Impairment due to pain (range 0–6)	− 0.77 (1.16)	− 0.93 (1.21)	− 0.42 (0.99)	1.643 (n.s.)
*Secondary outcome*					
Work ability					
WAI	Current work ability compared with lifetime best (range 0–10)	0.67 (2.04)	0.61 (2.33)	0.80 (1.20)	0.336 (n.s.)
	Work ability with respect to physical job demands (range 1–5)	0.28 (0.92)	0.27 (0.97)	0.30 (0.80)	0.109 (n.s.)
	Work ability with respect to mental job demands (range 1–5)	0.41 (0.68)	0.45 (0.73)	0.30 (0.57)	− 0.917 (n.s.)
	Work ability with respect to physical and mental job demands (range 2–10)	0.69 (1.32)	0.73 (1.40)	0.60 (1.14)	− 0.355 (n.s.)
Work-related, psychological well-being					
Irritation	Cognitive irritation (range 1–7)	− 0.90 (1.16)	− 0.88 (1.18)	− 0.93 (1.14)	− 0.173 (n.s.)
	Emotional irritation (range 1–7)	0.76 (0.87)	0.77 (0.92)	0.75 (0.77)	− 0.077 (n.s.)
	Overall irritation (range 1–7)	0.81 (0.84)	0.81 (0.88)	0.82 (0.78)	0.040 (n.s.)
MBI	Emotional exhaustion (range 1–6)	− 0.48 (0.87)	− 0.34 (0.90)	− 0.80 (0.74)	− 1.996*
	Depersonalisation (range 1–6)	− 0.28 (0.57)	− 0.30 (0.57)	− 0.23 (0.56)	0.453 (n.s.)

*E* external assessment, *FE* functional examination, *M* arithmetic mean, *MBI* Maslach Burnout Inventory, *MSC* musculoskeletal complaints, *NMQ* Nordic Musculoskeletal Questionnaire, *SD* standard deviation, *WAI* Work Ability Index, *WHYMPI* West Haven–Yale Multidimensional Pain Inventory;

*p = 0.05

As we did not observe significant differences between participants and drop-outs at baseline, we assumed that missing occurred at least at random and conducted multiple imputations, generating 20 complete data sets to analyse the intervention effects on MSC and on the secondary outcomes at the 3rd follow-up. Descriptive analyses of participants of the IG and CG at baseline and 3rd follow-up were performed with the non-imputed dataset (see Table 4).

**Table 4:** Descriptive statistics of the intervention outcomes at baseline and 3rd follow-up

		Group	Baseline	Follow-up	*M* (baseline)–*M* (follow-up)
			*M* (SD)	*M* (SD)	Δ (95% CI)
*Primary outcome*					
MSC					
Functional status of the locomotor system					
FE	Restriction of muscle strength (*E*) (range 0–12)	IG	4.88 (3.22)	3.79 (2.73)	1.08 (− 0.02; 2.18)
		CG	5.10 (2.63)	3.15 (2.77)	1.95 (0.77; 3.13)
	Restriction of maximum degree movement (*E*) (vertebral column) (range 0–24)	IG	6.50 (4.26)	3.33 (3.47)	3.17 (0.91; 5.42)
		CG	6.05 (4.98)	4.65 (4.44)	1.40 (− 0.93; 3.73)
NMQ	Restriction of everyday activities (last 3 months) (range 0–9)	IG	0.78 (1.31)	0.57 (1.20)	0.22 (− 0.44; 0.87)
		CG	1.45 (1.85)	0.50 (1.00)	0.95 (0.13; 1.77)
Pain severity/impairment due to pain					
FE	Pain due to maximum degree movement (range 0–210)	IG	20.09 (19.57)	6.70 (8.97)	13.14 (5.20; 21.07)
		CG	10.10 (9.78)	3.55 (5.10)	6.55 (2.75; 10.35)
WHYMPI	Pain severity on everyday movements (range 0–6)	IG	2.28 (1.33)	1.67 (1.47)	0.61 (0.10; 1.12)
		CG	2.35 (0.90)	1.55 (0.99)	0.80 (0.25; 1.35)
	Impairment due to pain (range 0–6)	IG CG	1.87 (1.44)	1.24 (1.51)	0.63 (0.10; 1.16)
			2.07 (1.26)	0.96 (1.08)	1.11 (0.63; 1.60)
*Secondary outcome*					
Work ability					
WAI	Current work ability compared with lifetime best (range 0–10)	IG CG	6.77 (2.43)	7.45 (2.20)	− 0.68 (− 1.73; 0.36)
			7.15 (1.23)	7.65 (1.95)	− 0.50 (− 1.47; 0.47)
	Work ability with respect to physical job demands (range 1–5)	IG CG	3.45 (0.67)	3.82 (0.96)	− 0.36 (− 0.81; 0.08)
			3.65 (0.67)	4.00 (0.56)	− 0.35 (− 0.70; − 0.00)
	Work ability with respect to mental job demands (range 1–5)	IG CG	3.50 (1.01)	4.05 (1.05)	− 0.55 (− 0.84; − 0.25)
			3.55 (0.83)	4.15 (0.67)	− 0.60 (− 1.07; − 0.13)
	Work ability with respect to physical and mental job demands (range 2–10)	IG CG	6.95 (1.40)	7.86 (1.81)	− 0.91 (− 1.50; − 0.31)
			7.20 (1.20)	8.15 (1.09)	− 0.95 (− 1.58; − 0.32)
Work-related. psychological well-being					
Irritation	Cognitive irritation (range 1–7)	IG CG	4.12 (1.50)	3.53 (1.61)	0.59 (0.12; 1.06)
			3.82 (1.78)	3.32 (1.55)	0.50 (− 0.24; 1.24)
	Emotional irritation (range 1–7)	IG CG	3.48 (1.50)	2.78 (1.52)	0.70 (0.31; 1.09)
			2.92 (1.51)	2.30 (1.07)	0.63 (− 0.02; 1.27)
	Overall irritation (range 1–7)	IG CG	3.72 (1.42)	3.06 (1.46)	0.66 (0.28; 1.04)
			3.26 (1.36)	2.69 (1.10)	0.57 (− 0.00; 1.13)
MBI	Emotional exhaustion (range 1–6)	IG CG	3.42 (1.33)	2.86 (1.33)	0.57 (0.14; 0.99)
			3.18 (1.02)	2.80 (0.96)	0.38 (− 0.17; 0.93)
	Depersonalisation (range 1–6)	IG CG	1.78 (0.93)	1.48 (0.90)	0.30 (0.07; 0.54)
			1.52 (0.62)	1.52 (0.66)	0.00 (− 0.33; 0.33)

*95% CI* 95% confidence interval, *CG* control group, *E* external assessment, *FE* functional examination, *IG* intervention group, *M* arithmetic mean, *MBI* Maslach Burnout Inventory, *NMQ* Nordic Musculoskeletal Questionnaire, *SD* standard deviation, *WAI* Work Ability Index, *WHYMPI* West Haven–Yale Multidimensional Pain Inventory

Then repeated measures ANOVA (see Table 5) was performed with and without imputations to analyse time and intervention effects.

**Table 5:** Analysis of intervention effects at 3rd follow-up

Outcome	Effect	Without imputation		20 imputations	
		*F*	*p*	*F*	*p*
Functional status of the locomotor system					
Restriction of muscle strength (*E*)	Group	0.072	0.789	1.025	0.312
	Time	15.231	0.000	11.568	0.001
	Group × time	1.243	0.271	0.489	0.485
Restriction of maximum degree movement (*E*) (vertebral column)	Group	0.176	0.677	0.177	0.674
	Time	8.485	0.006	5.525	0.019
	Group × time	1.270	0.266	4.907	**0.027**
Restriction of everyday activities (last 3 months)	Group	0.803	0.376	0.078	0.780
	Time	5.491	0.024	5.050	0.025
	Group × time	2.162	0.149	0.034	0.853
Pain severity/impairment due to pain					
Pain due to maximum degree movement	Group	4.629	0.038	5.122	0.024
	Time	20.358	0.000	23.090	0.000
	Group × time	2.279	0.139	3.432	0.064
Pain severity on everyday movements	Group	0.004	0.949	0.000	1.000
	Time	15.356	0.000	11.413	0.001
	Group × time	0.283	0.597	0.786	0.377
Impairment due to pain	Group	0.010	0.922	0.001	0.978
	Time	24.945	0.000	11.730	0.001
	Group × time	1.940	0.171	0.100	0.752
Work ability					
Current work ability compared with lifetime best (range 0–10)	Group	0.302	0.586	0.013	0.910
	Time	2.959	0.093	0.807	0.370
	Group × time	0.070	0.793	0.149	0.700
Work ability with respect to physical job demands (range 1–5)	Group	1.086	0.304	0.755	0.386
	Time	6.745	0.013	2.746	0.099
	Group × time	0.002	0.961	0.068	0.794
Work ability with respect to mental job demands (range 1–5)	Group	0.097	0.758	0.185	0.668
	Time	19.459	0.000	10.142	0.002
	Group × time	0.044	0.835	0.747	0.389
Work ability with respect to physical and mental job demands (range 2–10)	Group	0.482	0.491	0.332	0.565
	Time	19.903	0.000	6.316	0.013
	Group × time	0.010	0.922	0.021	0.884
Irritation					
Cognitive irritation (range 1–7)	Group	0.328	0.570	0.231	0.631
	Time	7.004	0.012	3.229	0.073
	Group × time	0.049	0.827	0.039	0.843
Emotional irritation (range 1–7)	Group	1.707	0.199	1.452	0.229
	Time	14.035	0.001	7.931	0.005
	Group × time	0.045	0.833	0.040	0.842
Overall irritation (range 1–7)	Group	1.188	0.282	0.784	0.376
	Time	14.584	0.000	6.655	0.010
	Group × time	0.084	0.773	0.267	0.606
MBI					
Emotional exhaustion	Group	0.205	0.653	0.111	0.740
	Time	8.271	0.006	5.695	0.018
	Group × time	0.304	0.584	0.433	0.513
Depersonalisation	Group	0.256	0.616	0.040	0.842
	Time	2.587	0.116	1.130	0.289
	Group × time	2.587	0.116	1.029	0.312

Results of repeated measures ANOVA with and without imputed datasets

*E* external assessment, *MBI* Maslach Burnout Inventory, group × time effects *p* < 0.05 are shown in bold

### Intervention effects on MSC

Significant time effects were found on all variables of the functional status of the locomotor system and pain in the imputed and non-imputed datasets. Restriction, pain severity and impairment due to pain decreased from baseline to the 3rd follow-up assessment in the IG and the CG.

#### Effects on the functional status of the locomotor system

A significant intervention effect was found on the restriction of maximum degree movement of the vertebral column for the imputed datasets only, which means that there was a greater decrease of impairments in the IG than in the CG at the 3rd follow-up. No further significant intervention effects were found.

#### Pain

The intervention effect on pain due to maximum degree movement was close to significance for the imputed datasets, which means that the decrease of pain tended to be stronger for the combined intervention in the IG compared to the CG in the 3rd follow-up. A significant group effect was further found on pain due to maximum degree movement for the imputed as well as for the non-imputed datasets, meaning that pain was, in general, lower in the CG than in the IG.

### Intervention effects on the secondary outcomes

#### Subjective work ability

Significant time effects were found on work ability with respect to mental job demands in the imputed as well as in the non-imputed datasets and for work ability with respect to physical job demands in the non-imputed dataset, meaning that work ability increased to the 3rd follow-up in the IG and the CG. However, no intervention effects were found.

#### Work-related psychological well-being

Furthermore, significant time effects were observed on irritation and the burnout dimension emotional exhaustion, such that irritation and emotional exhaustion decreased over time in the IG as well as in the CG. However, the effect on cognitive irritation was non-significant for the imputed datasets. In addition, no intervention effects were found on irritation or burnout.

## Discussion

Our study provided only marginal indications that the combination of a work-related individual psychosocial coaching intervention (stress management) with physiotherapy is in the long term (22 months after the end of the intervention) superior to the standard treatment (physiotherapy alone) to prevent unspecific MSC.

In only one outcome and only in the imputed dataset a statistically significant time × intervention group effect was observed: The restriction of maximum degree movement of the vertebral column decreased significantly more in the IG than in the CG. This is in accordance with our previous result that the IG showed a trend to a larger reduction of restriction of maximum degree movement compared to the CG immediately after the intervention (see Becker et al. 2017). This positive effect almost remained stable in the IG until 22 months after finishing the intervention with only a small reduction of effect size. In contrast, the former improvement of spinal mobility in the CG (Becker et al. 2017) declined nearly back to the baseline level during this phase of 22 months. To obtain information on functional status of the locomotor system, physical examination was performed by experienced physiotherapists who were blinded to the treatment group. Thus, a so-called “Hawthorne effect”, which means that the participants might have responded in a different way only because they knew that they were part of a intervention, cannot have influenced these results (see Levitt and List 2011).

With regard to the psychosomatic aetiology of MSC, psychological reactions to stressors might contribute to an increased tension of muscles and tendons, fasciae or ligaments (e.g. Campbell et al. 2013; da Costa and Vieira 2010; Gabbiani 1998; Gabbiani et al. 1972; Geurts and Sonnentag 2006; Griffiths et al. 2007; Hoogendoorn et al. 2000; Lang et al. 2012; Lundberg 2002; Sembajwe et al. 2013). Accordingly, previous studies indicate that psychosocial work stressors are related to the occurrence and persistence of MSC (e.g. Bernal et al. 2015; Campbell et al. 2013; da Costa and Vieira 2010; Hoogendoorn et al. 2000; Lang et al. 2012; Melloh et al. 2013; Sterud et al. 2014). Therefore, it is conceivable, that members of the IG used their learned stress management skills and were better able to cope with work stress over a long period of time. This might then have also contributed to an improvement of the functional status of the locomotor system, especially to an increase of vertebral movement.

In addition, regarding previous results on the immediate effects of the intervention, in the imputed dataset a trend of stronger pain reduction was observed in the IG than in the CG (Becker et al. 2017). Three and 22 months after the end of the intervention, pain intensity remained nearly consistent in both groups. Thus, after 22 months, members of the IG seemed to have a larger range of movement until pain occurs but not necessarily less pain than the CG. In contrast, the intervention effect at the 2nd follow-up on self-reported MSC outcomes like pain severity on everyday movements (Becker et al. 2017) disappeared 22 months later. Especially in the IG, participants reported that pain severity on everyday movements worsened after 22 months in comparison to the 2nd follow-up. The immediate findings at 2nd follow-up may, therefore, indicate a short-term effect of the intervention. However, also a short-term perceptual bias (“I tried hard, so the intervention must have done something.”) might have occurred and decreased after 22 months finishing the study.

Regarding restriction of muscle strength as well as everyday activities and impairment due to pain, no effects were observed, which corresponds to the findings after the 2nd follow-up (Becker et al. 2017). Since all participants were part of the active workforce, a healthy worker effect might have occurred. More specifically, restriction of muscle strength, everyday activities and impairment due to pain was already low at baseline and, therefore, intervention effects might have been more difficult to detect in our study sample.

Regarding MSC, the results of the presented study augment findings of Horneij et al. (2001), who observed that low back pain tended to improve up to 18 months following a work-place stress management intervention among home-care personnel compared to a control group. Our results complement those findings to the point that similar interventions might improve MSC (for up to 22 months after finishing the intervention) regarding a decrease of restrictions of spinal mobility.

In contrast to the 2nd follow-up (Becker et al. 2017)—where improvement of work ability and decrease of irritation and emotional exhaustion was more pronounced in the IG than in the CG—no long-term effects were observed on these outcomes at 3rd follow-up. Since the coaching intervention focused on strategies to reduce stress and to increase resources at work, this finding is surprising. One explanation might include the possibility that not all participants of the IG were able to achieve their self-set work-related goal to reduce work stress or to improve resources at work in the long run. As a result, these participants may not have been able to offset the imbalance between resources and work demands, which leads to reduced perceived work ability (see Tuomi et al. 2001; Tuomi et al. 1998). Consequently, frustration might have occurred and contributed to a deterioration of well-being after initial improvement. Moreover, the coaching intervention focused on one work stressor being personally relevant to the individual participants at the time of intervention and aimed to help participants to better cope with this one work stressor. At the time of the 3rd follow-up, these work stressors might have no longer been relevant to the participants and they might not have been able to transfer the coping strategies they acquired to new stressors. This, in turn, might have contributed to a decline of subjective well-being and work ability, independent of positive intervention effects on objective indicators of MSC. This points to the importance of continuous work-related coaching and training regarding work-specific stress management for nurses.

### Limitations and future research

The results point to the importance of continuous work-related coaching and training regarding work-specific stress management for nurses: (1) a statistically significant long-term intervention effect of the combined intervention was observed only on one out of four indicators of our primary outcomes. It, therefore, cannot be excluded that this single effect has occurred randomly. However, as stated above, there are plausible explanations for the observed differentiated effects that speak against such an assumption. Moreover, expert assessments by physiotherapists being blinded against the treatment allocation of participants exclude the risk of self-report bias. Nevertheless, future studies are needed to confirm potential additional effects of the psychosocial coaching intervention on MSC beyond the effects of physiotherapy alone. (2) It has to be acknowledged that the high number of drop-outs increases the risk of biased results and limits the power to detect intervention effects, which is a major limitation of the study. However, no differences in demographic and outcome variables at baseline were observed between drop-outs and participants. Moreover, to account for missing values and to improve the power of our analyses, we analysed 20 different simulated data sets that were completed with multiple imputation. Multiple imputation has been shown to produce unbiased parameter estimates under the assumption that data are missing at random (Schafer and Graham 2002). Unfortunately, it is impossible to test whether the data are missing or missing not at random since we have incomplete information on unobserved data. On the one hand, missing not at random might have occurred if participants were not able or refused to attend the follow-up examination due to low locomotor functioning, well-being, work ability or strong pain. On the other hand, especially participants with low locomotor functioning or strong pain might have preferred to attend the follow-up examination and data might rather be missing for healthier participants. Under both circumstances, missing would not be at random and multiple imputation could have led to biased results. (3) It is unclear whether participants of the IG and CG have continued to perform mobilizing physical exercises or participated in stress management interventions within the 22 months after finishing the intervention. Thus, different degrees of retention of training practices and different activities after the end of the intervention might have biased our findings. In further long-term studies, such information should be collected. (4) Finally, the single long-term intervention effect can only be attributed to the combined intervention (stress management intervention plus physical exercise) and not to a stress management intervention alone. Further studies should investigate which psychosocial mechanisms contribute in the long run to the prevention of MSC in the workplace.

## Conclusion

This study provides only marginal indications that a combined intervention of a work-related individual psychosocial coaching intervention with physiotherapy is in the long-term superior to the standard treatment (physiotherapy alone) to prevent unspecific MSC. There are indications that the combined intervention seems to be able to support the increase of the spinal mobility of the vertebral column. However, a clear long-term effect of the intervention on MSC could not be determined. Therefore, further studies are necessary to corroborate these findings.
